# Host immune responses and immune evasion mechanisms in *Trypanosoma cruzi*: implications for the immunopathogenesis of Chagas disease

**DOI:** 10.1590/0037-8682-0039-2026

**Published:** 2026-06-15

**Authors:** Caíco Mateus Pereira Andrade

**Affiliations:** 1 Centro Universitário Maurício de Nassau, Curso de Biomedicina, Petrolina, PE, Brasil.

**Keywords:** Chagas Disease, Immune response, Immune evasion, Cardiomyopathy, Public health

## Abstract

Chagas disease remains a major neglected tropical disease in Latin America and represents a significant public health challenge in Brazil. The progression of *Trypanosoma cruzi* infection is highly heterogeneous and reflects a complex interaction between parasite persistence and host immune responses. This narrative review summarizes current knowledge on host immune responses and immune evasion mechanisms during *T. cruzi* infection and discusses their implications for the immunopathogenesis and clinical progression of Chagas disease. The review included publications indexed in major biomedical databases, prioritizing studies on innate and adaptive immunity, parasite evasion strategies, and associations with chronic disease manifestations. Evidence indicates that innate immune mechanisms involving macrophages, dendritic cells, and natural killer cells are critical for early control of parasitemia through cytokine production, including interferon-gamma and tumor necrosis factor-alpha. Adaptive immunity, particularly CD4⁺ and CD8⁺ T lymphocytes, contributes to parasite control but may also promote chronic inflammation and tissue damage when regulatory mechanisms fail. In parallel, *T. cruzi* employs multiple immune evasion strategies, including complement inhibition, modulation of antigen-presenting cells, intracellular persistence, and antigenic variability, which facilitate long-term survival in host tissues. These interactions are central to chronic immunopathology, especially Chagas cardiomyopathy. Understanding these processes may contribute to improved diagnostic strategies, support identification of immunological biomarkers associated with disease progression, and inform advances in clinical management and public health approaches.

## INTRODUCTION

Chagas disease, caused by the protozoan parasite *Trypanosoma cruzi* (*T. cruzi*), is among the most important neglected tropical diseases in Latin America and remains a major public health challenge in Brazil^1^ ^-^ ^2^. Despite substantial advances in vector control and blood screening, millions of individuals remain chronically infected, and a considerable proportion develop severe clinical manifestations, particularly Chagas cardiomyopathy, with high morbidity and mortality^3^ ^-^ ^4^.

Brazil accounts for a substantial share of the global burden of Chagas disease, with marked regional heterogeneity and strong associations with socioeconomic vulnerability. Although triatomine vector transmission has been substantially reduced in several areas, oral transmission, congenital infection, and chronic cases diagnosed decades after exposure continue to sustain disease prevalence and impose a persistent burden on the Unified Health System (SUS)^5^ ^-^ ^6^.

In this context, understanding the biological mechanisms underlying disease progression is essential. Chagas disease progression is associated with distinct clinical forms and immunological profiles that evolve over time, directly influencing the transition from the indeterminate form to clinically manifest disease, particularly chronic Chagas cardiomyopathy^7^. Elucidating these pathways may support the identification of prognostic biomarkers, improve risk stratification, and guide targeted therapeutic and preventive strategies, thereby strengthening clinical management and informing public health policies.

The clinical course of *T. cruzi* infection is highly heterogeneous^8^. Following the acute phase, which is often oligosymptomatic or clinically mild, most individuals enter the indeterminate clinical form of Chagas disease, characterized by low but persistent parasitemia. Over time, approximately 30-40% of infected individuals progress to chronic symptomatic forms, most notably chronic Chagas cardiomyopathy, which is characterized by myocarditis, fibrosis, arrhythmias, and heart failure^8^.

Accumulating evidence indicates that variability in clinical outcomes is not determined solely by parasite burden, but by the dynamic and complex interaction between *T. cruzi* and the host immune system. Innate and adaptive immune responses play a crucial role in controlling parasite replication during early infection; however, they frequently do not achieve complete parasite clearance^9^. Persistent immune activation also contributes to chronic inflammation and progressive tissue damage, particularly in cardiac tissue^10^, directly affecting disease severity and clinical outcomes.

*T. cruzi* has evolved sophisticated immune evasion strategies that enable long-term persistence within host tissues. These mechanisms include modulation of inflammatory responses, complement evasion, interference with antigen presentation, and intracellular persistence in immune-privileged niches^11^ ^-^ ^12^. Together, these processes shape the immunopathogenesis of Chagas disease and directly influence its clinical progression.

Given the central role of host-parasite interactions in disease evolution, a comprehensive understanding of immune responses and immune evasion mechanisms is essential not only for advancing knowledge of Chagas disease immunopathogenesis but also for supporting improved diagnostic tools, therapeutic strategies, and evidence-based public health interventions to reduce disease burden.

## METHODS

This narrative literature review synthesized current knowledge on host immune responses and immune evasion mechanisms during *Trypanosoma cruzi* infection and their implications for the immunopathogenesis of Chagas disease.

A literature search was conducted in PubMed, Scopus, Web of Science, and SciELO for articles published up to 2026. The search strategy included combinations of the following terms: “Chagas disease”, “*Trypanosoma cruzi*”, “immune response”, “immune evasion”, “immunopathogenesis”, and “Chagas cardiomyopathy”.

Original research articles, review articles, and consensus guidelines published in English, Portuguese, or Spanish were considered. Studies involving humans were prioritized; however, experimental studies using animal models and in vitro systems were also included when relevant to clarify underlying immunological mechanisms.

Studies were selected according to their relevance to the following topics: innate and adaptive immune responses, immune evasion strategies of *T. cruzi*, parasite persistence, and immunopathogenesis associated with clinical progression of Chagas disease.

No formal systematic review protocol was followed, as the objective was to provide a narrative synthesis of the most relevant immunological and clinical evidence related to *Trypanosoma cruzi* infection. Efforts were made to include recent and relevant literature to ensure a comprehensive and balanced synthesis of current evidence.

## HOST IMMUNE RESPONSE TO *TRYPANOSOMA CRUZI*

### Innate immune response

The innate immune system constitutes the first line of defense against *T. cruzi* infection and is critical for the initial control of parasitemia. Shortly after infection, parasite-derived molecules are recognized by pattern recognition receptors (PRRs), including Toll-like receptors (TLRs), expressed on macrophages, dendritic cells, and other innate immune cells, as shown in experimental murine models, in vitro systems, and human studies^10^ ^-^ ^11^ ^,^ ^13^. This recognition triggers intracellular signaling pathways that promote the production of pro-inflammatory cytokines and antimicrobial mediators^13^.

During the acute phase, innate immune activation is characterized by rapid recognition of parasite molecules by Toll-like receptors (TLR2, TLR4, and TLR9), leading to activation of NF-κB signaling pathways and production of pro-inflammatory cytokines, including TNF-α, IL-12, and IFN-γ. These cytokines promote classical macrophage activation (M1 phenotype), enhancing nitric oxide production and parasite killing^9^ ^,^ ^11^.

In addition, monocytes and neutrophils contribute to early parasite control through phagocytosis and reactive oxygen species production. However, excessive activation of these pathways may contribute to early tissue damage and endothelial dysfunction^14^ ^-^ ^15^.

Macrophages play a key role in early host defense by phagocytosing the parasite and producing reactive oxygen species (ROS) and reactive nitrogen intermediates, particularly nitric oxide, which contribute to the killing of intracellular parasites. In parallel, dendritic cells process and present *T. cruzi* antigens, linking innate and adaptive immunity by activating T lymphocytes^11^.

Natural killer (NK) cells also contribute substantially to early immune control by producing interferon-gamma (IFN-γ), a key cytokine that enhances macrophage microbicidal activity and promotes a type 1 immune response. Experimental murine studies, supported by observations in patients with acute Chagas disease, have shown that IFN-γ is essential for limiting parasite replication during the acute phase of infection^13^.

Although these mechanisms reduce parasitemia, innate immune responses alone are insufficient to eradicate *T. cruzi*. The parasite can rapidly invade host cells and adapt to the intracellular environment, enabling it to evade early immune-mediated destruction and establish persistent infection^8^.

### Adaptive immune response

During the acute phase, adaptive immunity is marked by strong activation of CD8⁺ T cells and expansion of effector CD4⁺ Th1 cells. This response is associated with high production of IFN-γ and TNF-α, which is essential for parasite control. However, excessive or prolonged activation may initiate early immunopathological processes that contribute to tissue injury^12^ ^,^ ^16^.

The adaptive immune response is crucial for sustained control of *T. cruzi* infection and involves both CD4⁺ and CD8⁺ T lymphocytes. CD4⁺ T cells orchestrate immune responses through cytokine production, whereas CD8⁺ T cells are primarily responsible for cytotoxic elimination of infected host cells^9^ ^,^ ^16^. However, experimental studies and observations in patients with chronic Chagas disease, including clinical cohort studies, indicate that prolonged CD8⁺ T-cell activation, although essential for intracellular parasite control, may contribute to immune exhaustion and tissue injury during chronic infection^12^.

During acute infection, a strong type 1 adaptive immune response predominates, characterized by the production of IFN-γ and TNF-α, which is critical for parasite control but may contribute to immunopathology if sustained^16^.

CD8⁺ T cells recognize parasite-derived peptides presented by major histocompatibility complex (MHC) class I molecules, leading to targeted cytolysis. Although this mechanism is essential for controlling intracellular parasites, chronic activation is associated with tissue damage, particularly in cardiac muscle^11^.

Humoral immunity also plays a key role in *T. cruzi* infection. Specific antibodies contribute to parasite opsonization, neutralization, and complement activation; however, their effectiveness is partially limited by antigenic variability and parasite-mediated immune evasion mechanisms^15^ ^-^ ^16^.

### Key cytokines and immunological profiles

Cytokines are central mediators of the immune response to *T. cruzi* and strongly influence disease outcome, as demonstrated in both experimental models and human cohort studies. IFN-γ and TNF-α are essential for parasite control but are also implicated in chronic inflammation and tissue damage when dysregulated. Conversely, regulatory cytokines, such as interleukin-10 (IL-10) and transforming growth factor-beta (TGF-β), play a dual role by limiting excessive inflammation while potentially favoring parasite persistence^17^. These cytokine networks involving IFN-γ, TNF-α, and regulatory mediators, such as IL-10 help shape the balance between parasite control and immunopathology during chronic infection.

The balance between pro-inflammatory and regulatory cytokines determines the immunological profile of infected individuals and is closely associated with clinical progression. Patients with chronic Chagas cardiomyopathy often exhibit a persistent pro-inflammatory profile, whereas individuals with the indeterminate clinical form tend to display a more regulated immune response^11^ ^,^ ^18^. The main components of the host immune response involved in *Trypanosoma cruzi* infection are summarized in Table 1.

**TABLE 1:** Main components of the host immune response in *Trypanosoma cruzi* infection.

Immune component	Role in infection	Source of evidence	Clinical relevance
Macrophages	Phagocytosis and production of ROS and nitric oxide (NO), contributing to parasite killing	Animal, in vitro, human	Associated with early parasite control; dysregulation linked to chronic inflammation
Dendritic cells	Antigen processing and presentation; activation and polarization of T lymphocytes	Animal, in vitro, human	Impaired function may reduce effective adaptive immunity in chronic infection
Natural killer (NK) cells	Early production of IFN-γ and enhancement of macrophage microbicidal activity	Animal, human	Important for early parasitemia control; reduced activity may favor parasite persistence
CD4⁺ T lymphocytes	Coordination of immune responses via cytokine production (e.g., IFN-γ, TNF-α)	Human, animal	Imbalance associated with disease progression and immunopathology
CD8⁺ T lymphocytes	Cytotoxic elimination of infected cells via perforin/granzyme pathways	Human, animal	Chronic activation linked to myocardial damage and T-cell exhaustion
B lymphocytes	Differentiation into plasma cells and antibody production	Human, animal	Contribute to parasite control; dysregulation may affect chronic disease evolution
Antibodies (IgM, IgG)	Opsonization, neutralization, and complement activation	Human	Useful for diagnosis; limited efficacy due to parasite immune evasion

**ROS:** reactive oxygen species; **NO:** nitric oxide; **IFN-γ:** interferon-gamma; **TNF-α:** tumor necrosis factor-alpha. **Source:** Adapted from Tarleton^13^, Dutra et al.^11^, Medeiros et al.^9^, Macaluso et al.^16^.

### Immune evasion mechanisms of *Trypanosoma cruzi*

The ability of *T. cruzi* to establish long-term infection in the host is largely attributable to sophisticated immune evasion strategies. These mechanisms allow the parasite to survive host immune responses, persist within tissues, and drive chronic inflammation, ultimately contributing to disease progression. Immune evasion is therefore a central determinant of Chagas disease immunopathogenesis^11^ ^,^ ^13^.

### Complement system evasion

The complement system is one of the earliest host defense mechanisms encountered by *T. cruzi*. However, the parasite has developed multiple strategies to evade complement-mediated lysis. Surface molecules expressed by *T. cruzi*, such as complement regulatory protein (CRP) and decay-accelerating factor-like proteins, inhibit complement activation and prevent formation of the membrane attack complex (MAC), allowing the parasite to survive in extracellular environments during early infection^2^ ^,^ ^12^.

Resistance to complement-mediated killing is particularly relevant during the acute phase, when bloodstream trypomastigotes circulate and disseminate to target tissues. By escaping complement lysis, *T. cruzi* increases the likelihood of successful cell invasion and establishment of intracellular infection, setting the stage for chronic persistence^13^.

### Modulation of macrophages and dendritic cells

Beyond complement evasion, *T. cruzi* actively modulates the function of antigen-presenting cells, particularly macrophages and dendritic cells. Once inside these cells, the parasite interferes with intracellular signaling pathways, altering cytokine production and impairing effective antigen presentation^11^ ^-^ ^12^.

In macrophages, *T. cruzi* can suppress nitric oxide production and downregulate pro-inflammatory cytokines, thereby reducing microbicidal capacity. Additionally, the parasite influences macrophage polarization, favoring phenotypes that are less effective in parasite clearance and more permissive to intracellular survival^17^.

Dendritic cell dysfunction further compromises adaptive immunity. Impaired maturation and reduced expression of costimulatory molecules limit T-cell activation, resulting in suboptimal adaptive immune responses. Collectively, these effects weaken host defense mechanisms and facilitate parasite persistence^13^.

### Tissue persistence and intracellular survival

A defining feature of *T. cruzi* infection is its capacity for long-term intracellular persistence in a wide range of host tissues, particularly the myocardium and enteric nervous system. Once inside host cells, the parasite resides within intracellular compartments that protect it from circulating antibodies and complement-mediated lysis, thereby evading humoral immunity.

Persistent intracellular infection promotes chronic immune activation, even when parasite loads are low. This sustained antigenic stimulation drives prolonged inflammation and contributes to progressive tissue damage. Importantly, parasite persistence has been demonstrated in cardiac tissues of patients with chronic Chagas cardiomyopathy through molecular and histopathological techniques, including PCR detection of *T. cruzi* DNA in endomyocardial biopsy samples, supporting its central role in disease pathogenesis^12^ ^,^ ^19^.

In addition to intracellular persistence, *T. cruzi* employs other immune evasion mechanisms, including modulation of TLR signaling, interference with antigen presentation pathways, and induction of T-cell exhaustion, collectively impairing effective host immune responses.

The main immune evasion mechanisms employed by *T. cruzi* and their associated clinical implications are summarized in Table 2.

**TABLE 2:** Main immune evasion mechanisms of *Trypanosoma cruzi* and their clinical implications

Immune evasion mechanism	Description	Clinical implications	Source of evidence	Clinical relevance
Complement evasion	Expression of complement regulatory proteins inhibiting MAC formation	Increased parasite survival in bloodstream	In vitro, animal	Facilitates early infection and dissemination
Modulation of dendritic cells	Impaired maturation and reduced costimulatory molecule expression	Reduced T-cell activation	In vitro, animal, human	Contributes to ineffective adaptive immunity
Interference with antigen presentation	Disruption of MHC class I and II pathways	Impaired T-cell recognition	In vitro, animal	Limits cytotoxic and helper T-cell responses
TLR signaling modulation	Alteration of Toll-like receptor signaling pathways	Dysregulated innate immune responses	Animal, in vitro	Alters early immune activation and cytokine profiles
Cytokine modulation	Altered IFN-γ, TNF-α, IL-10 production	Chronic inflammation and immune imbalance	Human, animal	Associated with disease severity and progression
Intracellular persistence	Survival within host cells and immune-privileged niches	Chronic infection and sustained immune activation	Human, animal	Central mechanism driving chronic Chagas cardiomyopathy
Antigenic variability	Changes in parasite antigen expression	Impaired immune recognition	In vitro, animal	Hinders long-term immune clearance
T-cell exhaustion	Functional impairment due to persistent antigen exposure	Reduced parasite control	Human	Linked to chronic disease progression and severity
Evasion of cytotoxic T-cell responses	Reduced susceptibility to CD8⁺ T-cell-mediated killing	Persistence of infected cells	Animal, in vitro	Contributes to tissue damage and parasite survival

**MAC:** membrane attack complex; **IFN-γ:** interferon-gamma; **TNF-α:** tumor necrosis factor-alpha; **TLR:** Toll-like receptor. **Source:** Adapted from Cardoso et al.^15^, Dutra et al. ^11^, Pérez-Mazliah et al.^12^, Macaluso et al.^16^.

## CLINICAL PROGRESSION AND IMMUNOPATHOLOGY

### Acute versus chronic infection

The clinical progression of Chagas disease reflects a dynamic balance between host immune responses and parasite evasion strategies. During the acute phase, high parasitemia triggers strong innate and adaptive immune responses, including robust production of pro-inflammatory mediators, such as TNF-α and IFN-γ, which contribute to parasite control but may also induce endothelial dysfunction and early tissue injury^14^ ^,^ ^16^.

During the acute phase, *T. cruzi* also employs immune evasion strategies, including interference with antigen presentation and modulation of host immune signaling, which facilitates parasite survival and dissemination^15^.

As infection progresses to the chronic phase, parasitemia becomes low or undetectable in peripheral blood, but parasite persistence in tissues maintains chronic immune activation. Most individuals remain in the indeterminate clinical form, defined by positive serology for *T. cruzi* in the absence of clinical symptoms and without electrocardiographic or imaging evidence of cardiac or digestive involvement, according to established clinical classification criteria.

The indeterminate form is typically associated with a balanced immune response, characterized by controlled production of pro-inflammatory cytokines and increased regulatory mediators, such as IL-10, which limit tissue damage^11^ ^,^ ^18^.

In contrast, the cardiac form is associated with a persistent pro-inflammatory profile, with increased expression of cytokines, such as TNF-α, IFN-γ, and chemokines, including CCL2 and CXCL9, which promote recruitment of inflammatory cells to the myocardium and contribute to fibrosis and ventricular dysfunction^9^ ^-^ ^10^.

The digestive form is characterized by destruction of the enteric nervous system, inflammatory infiltrates, and altered neuronal regulation, although its immunological profile is less well defined than that of the cardiac form^8^.

These symptomatic cardiac and digestive manifestations reflect differences in host immune regulation and inflammatory profiles^16^.

Recent longitudinal studies have shown that immune responses during the acute phase, particularly in orally transmitted infections, can influence long-term immunological profiles and clinical outcomes, even after antiparasitic treatment^20^.

### Chagas cardiomyopathy

Clinical studies in Brazilian cohorts have demonstrated that patients with chronic Chagas cardiomyopathy exhibit a predominantly pro-inflammatory cytokine profile, characterized by elevated IFN-γ and TNF-α levels, whereas individuals with the indeterminate form display a more regulated immune response with higher IL-10 production^10^ ^,^ ^18^.

Chronic Chagas cardiomyopathy represents the most severe manifestation of *T. cruzi* infection and is characterized by myocarditis, fibrosis, ventricular remodeling, arrhythmias, and progressive heart failure. Immunopathological mechanisms play a central role in this process, particularly the persistence of low-level parasitism combined with chronic immune activation^11^ ^-^ ^12^ ^,^ ^21^.

Chemokines play a central role in leukocyte recruitment to cardiac tissue. Increased expression of chemokines, such as CCL2, CCL5, and CXCL9 has been observed in patients with chronic Chagas cardiomyopathy and is associated with inflammatory cell infiltration and disease severity^9^ ^-^ ^10^.

In addition, fibrotic mediators, such as transforming growth factor-beta (TGF-β) contribute to myocardial fibrosis and ventricular remodeling. Cytotoxic mechanisms involving perforin and granzyme released by CD8⁺ T cells further contribute to cardiomyocyte damage^21^ ^-^ ^22^.

Elevated pro-inflammatory mediators, including TNF-α and IFN-γ, along with chemokines and cytotoxic T-cell responses, are associated with myocardial inflammation, fibrosis, and electrical conduction abnormalities. In addition, endothelial dysfunction and microvascular alterations further contribute to cardiac damage^14^ ^,^ ^16^.

### Immunological-clinical correlation

The correlation between immune profiles and clinical outcomes is a defining feature of Chagas disease. Patients with indeterminate forms typically exhibit more balanced immune responses, characterized by effective parasite control with limited tissue damage. In contrast, individuals with cardiomyopathy display persistent pro-inflammatory profiles, increased expression of T-cell exhaustion markers, and impaired regulatory mechanisms^18^.

Several human cohort studies have investigated the relationship between immune responses and clinical forms of Chagas disease. In Brazilian populations, patients with indeterminate forms typically exhibit balanced immune responses with increased regulatory cytokines, whereas those with cardiomyopathy show sustained pro-inflammatory profiles and markers of T-cell exhaustion. These findings highlight the role of immune regulation in determining disease progression and clinical outcomes^10^ ^,^ ^18^.

**FIGURE 1:**
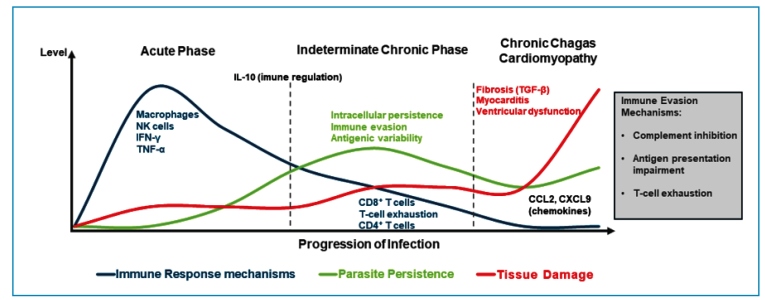
Immunopathogenesis of Chagas disease progression. Schematic representation of the dynamic interaction between host immune response, parasite persistence, and tissue damage during the progression of *Trypanosoma cruzi* infection. During the acute phase, strong innate and adaptive immune responses (e.g., macrophages, NK cells, IFN-γ, TNF-α) contribute to parasite control. In the indeterminate chronic phase, immune regulation (e.g., IL-10) helps limit tissue damage despite persistent infection. In contrast, chronic Chagas cardiomyopathy is associated with sustained inflammation, chemokine-mediated leukocyte recruitment (e.g., CCL2, CXCL9), T-cell exhaustion, and fibrotic processes (e.g., TGF-β), leading to myocardial damage and ventricular dysfunction. Immune evasion mechanisms, including complement inhibition, antigen presentation impairment, and T-cell exhaustion, contribute to parasite persistence and disease progression.

**Source:** Adapted from Dutra et al.^11^; Cunha-Neto et al.^10^; Medeiros et al.^9^; Pérez-Mazliah et al.^12^; Ramasawmy and Cunha-Neto^22^.

These findings support the concept that Chagas disease progression is driven not only by parasite persistence but also by the quality and regulation of host immune responses over time (Figure 1)^9^ ^-^ ^10^ ^,^ ^16^.

## IMPLICATIONS FOR CLINICAL MANAGEMENT AND PUBLIC HEALTH

The immunopathogenic mechanisms underlying Chagas disease have direct and actionable implications for clinical management and health system organization, particularly in endemic settings, such as Brazil. A clearer understanding of host immune responses and parasite evasion strategies enables translation of immunological knowledge into practical tools for disease control^9^ ^,^ ^16^.

These findings demonstrate that immunological mechanisms are not only relevant to pathogenesis but also directly inform surveillance strategies, risk stratification, and resource allocation within the Brazilian health system.

### Diagnosis

Advances in immunology have enabled the identification of biomarkers associated with disease progression, including cytokine profiles (e.g., IFN-γ, TNF-α), chemokines (e.g., CCL2, CXCL9, CXCL10), and markers of T-cell activation and exhaustion. In human studies, elevated levels of pro-inflammatory cytokines and chemokines, together with reduced regulatory responses, such as IL-10, have been associated with myocardial fibrosis, ventricular dysfunction, and increased risk of progression to cardiomyopathy^9^ ^-^ ^10^ ^,^ ^18^. These biomarkers may support risk stratification, early detection of cardiac involvement, and clinical decision-making (Table 3).

**TABLE 3:** Immunological biomarkers associated with clinical progression and severity in Chagas disease

Biomarker	Immunological role	Clinical association	Source of evidence
IFN-γ	Pro-inflammatory cytokine; macrophage activation and parasite control	Elevated in cardiac form; associated with myocardial inflammation	Human
TNF-α	Pro-inflammatory cytokine; promotes inflammation and endothelial activation	Associated with cardiomyopathy severity and ventricular dysfunction	Human
IL-10	Regulatory cytokine; limits excessive inflammation	Higher levels in indeterminate form; associated with controlled disease	Human
CCL2 (MCP-1)	Chemokine that recruits monocytes/macrophages	Increased in cardiac patients; linked to myocardial inflammation	Human
CXCL9 (MIG)	Chemokine induced by IFN-γ; recruits T cells	Associated with cardiac inflammation and disease progression	Human
TGF-β	Fibrotic mediator; promotes extracellular matrix deposition	Linked to myocardial fibrosis and ventricular remodeling	Human, animal
Perforin / Granzyme B	Cytotoxic molecules released by CD8⁺ T cells	Associated with cardiomyocyte damage in chronic infection	Human, animal
CD8⁺ T-cell exhaustion markers (e.g., PD-1)	Indicators of T-cell dysfunction due to chronic antigen exposure	Associated with disease progression and reduced parasite control	Human
IL-6	Pro-inflammatory cytokine involved in acute phase response	Associated with inflammation and cardiac dysfunction	Human
CXCL10 (IP-10)	Chemokine that recruits activated T cells	Correlates with cardiac disease severity	Human

**Source:** Adapted from Cunha-Neto et al.^10^, Medeiros et al.^9^, Costa et al.^18^, Ramasawmy and Cunha-Neto^22^, Macaluso et al.^16^.

### Treatment

Current antiparasitic therapies, such as benznidazole, are most effective during the acute phase but show limited efficacy in chronic infection. Parasite persistence and chronic immune activation contribute to these treatment limitations. Recent evidence suggests that combining antiparasitic drugs with immunomodulatory approaches may improve outcomes by targeting both parasite burden and immune dysregulation^11^ ^-^ ^12^ ^,^ ^23^.

Host immune response also influences treatment outcomes. Patients with a more regulated immune profile may respond better to antiparasitic therapy, whereas persistent inflammation and immune dysregulation are associated with reduced treatment efficacy and disease progression^9^ ^,^ ^12^.

### Vaccines: challenges and perspectives

Vaccine development remains challenging because of antigenic variability, parasite immune evasion, and the need to avoid exacerbating immunopathology. Mechanisms, such as interference with antigen presentation, dendritic cell modulation, and T-cell exhaustion complicate the induction of effective protective immunity. Understanding these mechanisms is essential for the rational design of vaccine candidates^15^ ^-^ ^16^ ^,^ ^24^.

Effective vaccine strategies must balance induction of protective Th1-type responses with control of excessive inflammation. The ability of *T. cruzi* to induce T-cell exhaustion and modulate antigen presentation remains a major challenge for vaccine development^15^ ^,^ ^24^.

### Relevance for the Brazilian Unified Health System (SUS)

Chagas disease imposes a substantial long-term burden on the Brazilian Unified Health System (SUS), particularly through chronic cardiac and digestive complications that require prolonged and specialized care^5^ ^-^ ^6^. This burden is especially relevant in endemic regions, where delayed diagnosis and heterogeneous clinical progression challenge health system efficiency.

Advances in immunopathogenesis provide opportunities to improve health system strategies. Immunological biomarkers may enhance surveillance by enabling risk stratification and identifying patients at higher risk of disease progression^9^ ^,^ ^18^. Incorporating immune markers into clinical protocols may also support earlier detection of cardiac involvement and guide therapeutic decision-making.

Furthermore, understanding the role of persistent inflammation and immune dysregulation supports the development of targeted therapies, which may reduce disease progression, hospitalizations, and long-term healthcare costs^12^ ^,^ ^16^.

Thus, integrating immunological insights into surveillance, diagnosis, and treatment strategies can strengthen health system responses and improve long-term outcomes in endemic regions.

This integration may support cost-effective strategies within the SUS by prioritizing high-risk patients and optimizing resource allocation.

## CONCLUSION AND FUTURE PERSPECTIVES

Chagas disease progression is driven by a complex interplay between host immune responses and immune evasion strategies employed by *T. cruzi*. Although immune mechanisms are essential for parasite control, their dysregulation contributes to chronic inflammation and tissue damage, particularly in the heart^11^ ^-^ ^12^.

Advances in understanding immunopathogenesis provide important translational opportunities. Identifying immunological biomarkers may improve early diagnosis and risk stratification, while new therapeutic approaches targeting both parasite persistence and immune dysregulation may enhance clinical outcomes^9^ ^,^ ^16^.

In Brazil, integrating immunological knowledge into public health strategies within the SUS framework is essential for reducing disease burden, improving early intervention, and optimizing long-term patient care^5^ ^-^ ^6^.

## Data Availability

This study is a narrative review based exclusively on published literature, and no primary research data were generated or analyzed.
